# Quantitative Approach to Quality Review of Prenatal Ultrasound Examinations: Fetal Biometry

**DOI:** 10.3390/jcm13164860

**Published:** 2024-08-17

**Authors:** C. Andrew Combs, Sushma Amara, Carolyn Kline, Olaide Ashimi Balogun, Zachary S. Bowman

**Affiliations:** 1 The Pediatrix Center for Research, Education, Quality & Safety, Sunrise, FL 33323, USA; 2 Obstetrix of California, Campbell, CA 95008, USA; 3 Eastside Maternal-Fetal Medicine, Bellevue, WA 98004, USA; 4 Obstetrix Maternal-Fetal Medicine Specialists, Houston, TX 77054, USA; 5 Perinatal Associates of Sacramento, Sacramento, CA 95816, USA

**Keywords:** abdominal circumference, femur length, head circumference, image review, quality review, variation, z-score

## Abstract

**Background/Objectives**: To evaluate the quality of an ultrasound practice, both large-scale and focused audits are recommended by professional organizations, but such audits can be time-consuming, inefficient, and expensive. Our objective was to develop a time-efficient, quantitative, objective, large-scale method to evaluate fetal biometry measurements for an entire practice, combined with a process for focused image review for personnel whose measurements are outliers. **Methods**: Ultrasound exam data for a full year are exported from commercial ultrasound reporting software to a statistical package. Fetal biometry measurements are converted to z-scores to standardize across gestational ages. For a large-scale audit, sonographer mean z-scores are compared using analysis of variance (ANOVA) with Scheffe multiple comparisons test. A focused image review is performed on a random sample of exams for sonographers whose mean z-scores differ significantly from the practice mean. A similar large-scale audit is performed, comparing physician mean z-scores. **Results**: Using fetal abdominal circumference measurements as an example, significant differences between sonographer mean z-scores are readily identified by the ANOVA and Scheffe test. A method is described for the blinded image audit of sonographers with outlier mean z-scores. Examples are also given for the identification and interpretation of several types of systematic errors that are unlikely to be detectable by image review, including z-scores with large or small standard deviations and physicians with outlier mean z-scores. **Conclusions**: The large-scale quantitative analysis provides an overview of the biometry measurements of all the sonographers and physicians in a practice, so that image audits can be focused on those whose measurements are outliers. The analysis takes little time to perform after initial development and avoids the time, complexity, and expense of auditing providers whose measurements fall within the expected range. We encourage commercial software developers to include tools in their ultrasound reporting software to facilitate such quantitative reviews.

## 1. Introduction

Obstetrical ultrasound diagnosis depends on highly skilled personnel to obtain images and interpret the findings. Fetal measurements are typically obtained by sonographers and reviewed and interpreted by sonologists (physicians). There is an inherent potential for diagnostic error because people are not perfect. Although there are well-defined standards for correct image planes and correct caliper placement for fetal biometry, sonographers have varying levels of skill in obtaining correct measurements. Some may systematically place the calipers too widely or may frequently measure in oblique planes, resulting in overmeasurement. Others may systematically place the calipers too narrowly, resulting in undermeasurement.

Measurement errors can have clinical consequences. The basic fetal biometry measurements, biparietal diameter (BPD), head circumference (HC), abdominal circumference (AC), and femur length (FL), are used to calculate an estimated fetal weight (EFW). Although EFW is known to differ from birth weight by more than 10% in one quarter of exams [1,2], EFW and its percentile are often used to make clinical decisions. When EFW or AC are less than the 10th percentile, a diagnosis of fetal growth restriction (FGR) is made and follow-up is recommended, including fetal surveillance, a repeat assessment of fetal growth, and sometimes preterm or early-term delivery [3]. When EFW is large, the induction of labor is sometimes recommended and the risk of cesarean is increased, even if the actual birth weight is not increased [2,4,5,6,7,8,9,10,11].

**Figure 1:**
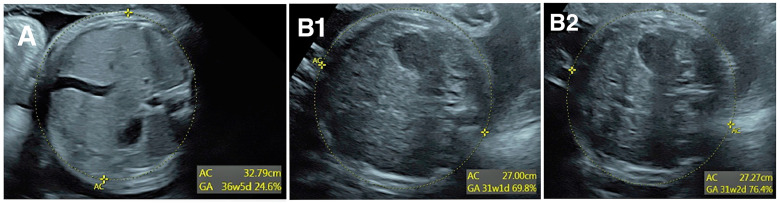
Abdominal circumference (AC) measurements shown by yellow dotted ellipses. In panel (**A**), AC is clearly undermeasured. In panels (**B1**,**B2**), the difference in AC is only 1%, but systematic differences of this magnitude can be clinically relevant.

The interpreting physician is the first step in detecting and correcting measurement error, but this first level review does not prevent all errors. Consider the three images of AC in Figure 1. In panel A, the calipers are placed far inside the fetal abdomen, so AC is clearly undermeasured. Though few providers would find this image acceptable, it was obtained in a very busy ultrasound practice and the measurement error was not detected by the sonographer or the reading physician. Panels B1 and B2 show two images from a different fetus taken a few seconds apart. An important limitation is how difficult it is to see the ellipses measuring AC. Notwithstanding that difficulty, the difference in AC between panels B1 and B2 is 2.7 mm or 1%, with AC slightly undermeasured in panel B1. Although this might seem inconsequential, the standard deviation (SD) of AC is 13.4 mm per Hadlock et al. [12], so 2.7 mm is 0.2 SD. If a sonographer systematically undermeasures AC by 0.2 SD, they will find AC < 10th percentile in 14% of exams rather than the expected 10%. This would result in a 40% overdiagnosis of fetal growth restriction, which in turn would result in excess costs of fetal surveillance, repeat exams, and patient anxiety; yet a systematic undermeasurement of this magnitude would probably go undetected.

Systematic quality review is recommended by various professional organizations to assure the accuracy of obstetrical ultrasound diagnoses [13,14,15,16]. Accreditation by the American Institute of Ultrasound in Medicine [13] requires that a practice “must show ongoing monitoring of the clinical practice’s personnel performance, including all physicians and sonographers through regular, retrospective review. A record of quality assurance (QA) activities must be maintained and kept current”. The Society for Maternal–Fetal Medicine (SMFM) [16] states that “optimal QA monitoring includes large-scale audits and focused audits and should be used to provide constructive individual and group feedback”. 

In a typical quality audit, an expert supervisor or peer will examine a sample of images obtained by a given provider, looking for proper image planes and caliper placement. However, an image review has several limitations:

- Image review is labor-intensive, requiring unreimbursed personnel time taken away from clinical care [15].
- The majority of images from the majority of providers meet quality standards, so a large number of images must be reviewed to identify the occasional outlier [17,18].
- Image review is somewhat subjective and prone to biases, especially if the auditor knows the identity of the provider being reviewed [19].
- There are no evidence-based standards to guide how often reviews should be performed or how many exams should be selected to ensure a representative sampling.

To address the limitations of an image review, we have developed objective, quantitative methods to evaluate the findings of individual sonographers and physicians in our maternal–fetal medicine (MFM) practices. This article details our methods for a quality review of fetal biometry measurements. Subsequent articles will address quantitative methods for the quality review of fetal anatomy surveys and EFW.

## 2. Materials and Methods

Our approach to biometry review was modeled after the Nuchal Translucency Quality Review (NTQR) program of the Perinatal Quality Foundation [20]. For the NTQR program, practices submitted limited data for review: exam date, crown-rump length (CRL), nuchal translucency (NT) measurement, and identifier codes for sonographer and physician. For each exam, the NTQR program calculated the difference between the observed NT and the expected value of NT for the given CRL. The program then summarized those differences and sent a quarterly report to each participating provider indicating whether the NTs they obtained were larger or smaller than expected, on average, and whether the variance was within the expected range. In this way, the NTQR program identified providers whose measurement technique needed scrutiny even though *providers did not send any images for review*.

Our MFM practices use Viewpoint software (Version 6, GE Healthcare, Wauwatosa, WI, USA) to store exam data and generate reports. The software includes a query tool to extract specific data from all exams meeting specified criteria. For each practice, we extract a full calendar year of data for all exams that have measurements of AC, biparietal diameter (BPD), head circumference (HC), and femur length (FL). In addition to the measurements, we extract the exam date, gestational age (GA), sonographer name, reading physician name, fetal cardiac activity (present or absent), plurality (singleton, twin, triplet, etc.), and exam status (final, revised, incomplete, etc.). Viewpoint generates a data file in comma-separated value format (.csv file), with one row per exam and one column for each data field. We open the .csv file in Excel (Version 15, Microsoft, Redmond, WA, USA) and save it as an Excel Workbook (.xlsx file). We import the Excel file into Stata statistical software (Version 13, Statacorp, College Station, TX, USA). The file in the Supplementary Materials shows an example of the Stata script used for the analysis.

Inclusion criteria for the quality audit are as follows: finalized (signed) exam, fetal cardiac activity present, singleton pregnancy, and GA from 14^0/7^ to 39^6/7^ weeks. Exams not meeting these criteria are excluded from analysis. Our practices used GE Voluson or Philips Affiniti (Philips Electronics, Amsterdam, Netherlands) ultrasound machines, with multifrequency transducers ranging from 2 to 10 mHz as needed to optimize imaging. 

To standardize the measurements across GAs, we calculate the z-score for BPD, HC, AC, and FL for each exam. The z-score is the number of SDs a measurement falls above or below the expected mean, based on a standard or reference norm. We use the Hadlock references [12] for BPD, HC, AC, and FL, because those are the norms used for on-screen displays during the exams and in the reports from our practices. If a reference or standard is a perfect fit for a sample of measurements, the z-scores will be normally distributed with a mean of 0 and an SD of 1. Extreme outliers, defined as observations more than 6 SD from the mean (i.e., z-score < −6 or z-score > 6), are excluded from the quantitative audit because of their potential to skew the mean, but they are audited individually by comparing images to reported values. We summarize each provider’s measurements by calculating their mean and SD of z-score. 

Differences between providers are tested with one-way analysis of variance (ANOVA) and Scheffe test. Two-tailed *p*-values < 0.05 are considered significant. We present example scatterplots and histograms to illustrate certain points, but the figures are not a routine part of the quality reviews.

A focused image review is performed for providers whose z-scores differ significantly from their colleagues. The procedure for blinded image review of a randomly selected subset of exams is detailed in a later section.

We have used this method for quality audits at 8 MFM practices in our national MFM group since 2019. The results in the figures and tables below are actual data from a subset of sonographers and physicians at one of our AIUM-accredited practices. The sonographers were all certified by the Registry of Diagnostic Medical Sonographers and the physicians were all certified in MFM by the American Board of Obstetrics and Gynecology. The results presented are typical examples of the findings in our complete audits, although the providers have been hand-selected to illustrate some of the issues that can be identified by a quantitative audit. To protect their confidentiality, we do not reveal individual names, practice location, or the year of the exams. In the tables and figures that follow, we summarize an analysis of fetal AC. Analogous methods can be used for BPD, HC, and FL.

## 3. Results

### 3.1. Sonographer Mean z-Score Values

**Figure 2:**
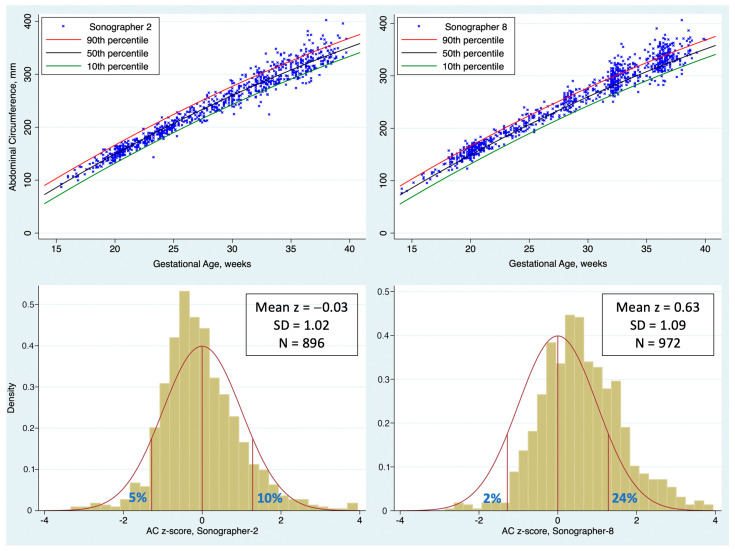
Abdominal circumference (AC) measurements by two sonographers over one year. Upper panels are scatterplot of AC across gestational age, along with 10th, 50th, and 90th percentiles for AC based on Hadlock reference [12]. Lower panels are histograms of the AC z-score for the same observations. The red curve shows the ideal distribution based on Hadlock reference, with droplines at 10th, 50th, and 90th percentiles (left-to-right, respectively). The blue numbers in the tails show the percent of observations <10th percentile and >90th percentile (left and right, respectively).

Figure 2 shows one year of AC measurements from two sonographers in the same practice. The upper panels are scatterplots of the AC measurements across GA. A z-score is calculated for each measurement to standardize the observations at different GAs. The lower panels show histograms of the z-score for the two sonographers. Sonographer 2 (left panel) had a mean z-score of −0.03 (not significantly different than 0) and an SD of z-score very close to 1, indicating an excellent fit to the Hadlock reference [12]. In contrast, the measurements of Sonographer 8 (right panel) are shifted far to the right, with a mean z-score of 0.63. This resulted in a paucity of exams with AC < 10th percentile (2.4% compared to the expected 10%) and an excess of exams with AC > 90th percentile (24% compared to the expected 10%). 

Table 1 summarizes the mean and SD of the AC z-score for eight sonographers from the same practice for one year. For the whole practice, the mean z-score was 0.33, meaning that the practice overall tends to find AC about 1/3 of an SD (4.4 mm) larger than expected based on the Hadlock reference [12]. There are several possible explanations for this: the practice may have a high percentage of patients with obesity, diabetes, and other risk factors for large-for-gestational age (LGA) fetuses; the sonographers at this practice may have been trained to place their calipers slightly outside the fetal abdomen; a high percentage of exams may have been oblique cross-sections rather than perpendicular sections; or the Hadlock reference [12] may have values that are too small for the modern US population. The data in hand do not permit a simple explanation for this deviation. If it is simply a reflection of a population enriched for LGA, it may not be an issue at all.

However, *variations between sonographers within the practice* cannot be attributed to any of these explanations. If sonographers examine a random selection of patients, it is expected that they should all have approximately the same distribution of z-scores. Yet, Sonographers 1 and 2 had mean z-scores very close to zero while Sonographers 6, 7, and 8 had a means > 0.50, that is, over half an SD larger than expected. The impact of these differences is illustrated in the right two columns of Table 1. Sonographers 6–8 all had a paucity of exams < 10th percentile (3% of exams or fewer, compared to the expected 10%) and an excess of exams > 90th percentile (20% of exams or more, compared to the expected 10%).

The ANOVA shows a significant overall difference between sonographer mean z-scores (footnote g, *p* < 0.001). The Scheffe multiple comparisons tests demonstrate significant pairwise differences between sonographers (footnotes b through e, all *p* < 0.02). 

### 3.2. Image Audit Focused on Outliers

Focused image audits are recommended for sonographers whose measurements are outliers. There is little value in auditing those whose measurements lie close to the practice mean, because the majority of their images will be within accepted standards. For the practice illustrated in Table 1, we recommended audits for Sonographers 1, 2, and 8 because their mean z-scores had the largest deviations from the practice mean. 

Image audits are performed anonymously, i.e., the auditor does not know the identity of the persons being audited and does not know whether their measurements are, on average, larger or smaller than expected. For this reason, it is ideal to simultaneously audit at least one sonographer whose mean is below the practice mean (possible systematic undermeasurement) and one whose mean is above the practice mean (possible systematic overmeasurement). At least two people are needed to perform a blinded review, one to select and prepare the images and another to evaluate them. If there are adequate personnel and time, an additional blinded auditor evaluates the same images. These tasks are performed by the lead sonographer, the physician ultrasound director, and other practice leaders as available.

A sample of 15–20 exams per sonographer is usually sufficient to identify systematic overmeasurement (oblique planes or calipers consistently placed too widely) or undermeasurement (calipers placed too narrowly). The example Stata script in the Supplementary Materials includes a section for generating a random subset of 20 exams for two sonographers selected for an audit. If statistical software is not used, then an arbitrary set of recent exams by that sonographer can be selected manually.

Most ultrasound image storage systems allow for the export of anonymized images. For each exam selected for audit, we anonymize and export all the images showing AC measurements. The person preparing the images keeps a key with exam identifiers and sonographer identifiers, but the key is not shared with the auditors. The selected images are compiled in a computer file folder and shared with the auditors. We review the image files on a computer monitor rather than printing them on paper for two reasons: first, printed images are generally of lower quality; and second, the original exam and original interpretation are performed via monitors, not on paper.

Auditors keep a scoresheet, comparing the images for each exam to the reported value, judging whether the reported value represents overmeasurement, undermeasurement, or acceptable measurement, and recording any notes about improper image planes. Once the scoring is completed, the key is opened and each sonographer’s scores are compiled.

If the majority of images from a sonographer are rated as over- or undermeasurement, and especially if this matches the expected result based on the z-score, this is discussed privately with the sonographer. Sonographers often feel threatened by a quality audit and their privacy must be respected. The discussion is one-on-one, conducted by the lead sonographer or practice ultrasound director, depending on who the sonographer will likely find least threatening. The discussion emphasizes that the process is not intended to be punitive but rather to identify opportunities to improve measurement technique. The sonographer is told that the practice routinely monitors the measurement of all sonographers on an ongoing basis and that this sonographer was identified as an outlier. Any constructive suggestions about improving technique are discussed

If the majority of images from a sonographer are rated as acceptable, this is also shared with the sonographer. In a one-on-one session, the sonographer is told that they were identified as a possible outlier in the routine monitoring of measurements, but that review of their images did not find a systematic issue.

### 3.3. Evaluation of Standard Deviation (SD) of z-Score

**Figure 3:**
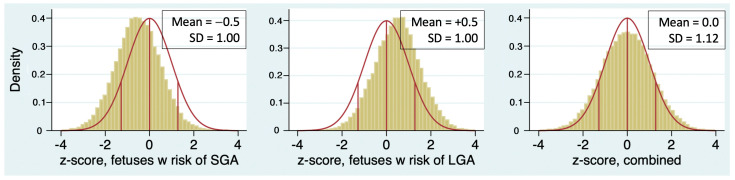
Simulation showing that combining two samples with standard deviation (SD) equal to 1 will result in a population with SD > 1.

In Table 1, the SD of z-score is greater than 1 for every sonographer except Sonographer 1. The population of patients undergoing ultrasound in a typical MFM practice is usually enriched with patients at risk for both LGA fetuses (e.g., diabetes, obesity) and small-for-gestational age (SGA) fetuses (e.g., hypertensive disorders, suspected growth restriction, advanced maternal age). The consequence of this is that the SD of z-score should be greater than 1, as illustrated in Figure 3. It is unlikely that a sonographer examining a random selection of patients from a mixed-risk population will have an SD less than 1.

**Figure 4:**
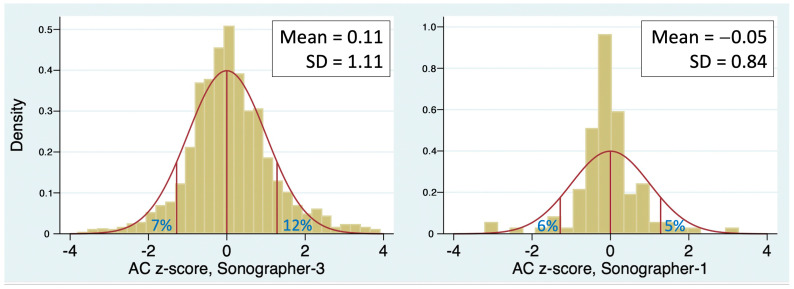
Abdominal circumference (AC) z-scores of two sonographers (bars) with similar means but different standard deviations (SD). When SD is <1 (right panel), there is an excess of exams with z-score near 0 and a paucity of exams in both tails, likely resulting from “expected-value bias”.

When the SD of z-score is <1, a likely explanation is “expected-value bias”, which occurs when a sonographer adjusts the caliper placement to make the measurement match the gestational age displayed on the screen [21]. The observation that Sonographer 1 had both an SD < 1 and a mean z-score very close to zero suggests that this is occurring (Table 1). Sonographer 1 also had SD < 1 for the HC and FL z-scores (0.90 and 0.76, respectively), reinforcing the notion that expected-value bias may be occurring. The problem with expected-value bias is that forcing the measurements to be “normal” will result in missed diagnoses of large or small measurements, that is, fewer than 10% of measurements will be <10th percentile or >90th percentile, as illustrated in Figure 4. 

Expected-value bias generally cannot be detected by an image review. If it is suspected, as with Sonographer 1, our approach is to discuss the findings with the sonographer involved and ask whether their customary process is to “fine-tune” their measurements to match the gestational age. If so, it may be sufficient to give a brief educational intervention regarding why this should be avoided. A follow-up audit will determine whether the issue has been corrected.

Sonographer 7, on the other hand, had an SD much larger than the other sonographers (Table 1). This suggests inconsistency in measurement, that is, the sonographer sporadically both overmeasures and undermeasures AC. This possibility is reinforced by the observation that Sonographer 7 also had a larger SD of z-scores for both HC and FL than all the other sonographers. In such cases, we recommend mentoring on taking greater care in caliper placement. We also note that Sonographer 7 had a relatively small number of exams, so it is possible that the large SD might be spurious. A follow-up audit will reveal whether the issue is persistent.

### 3.4. Physician Mean Values

Table 2 shows the mean and SD of z-score for five physicians from the same practice as those in Table 1. In our practices, the physicians generally read and interpret exams performed by sonographers, and rarely perform the primary measurements themselves. Thus, differences between physicians likely reflect differences in the sonographers whose exams they interpret, rather than different measurements by the physicians.

To adjust for the variance between sonographers, a formal multivariable regression can be used, but this is a complex task that will usually require professional statistical consultation. Instead, we perform a simpler adjustment: for each exam, we subtract the mean z-score of the sonographer who performed the exam. As shown in the right-hand section of Table 2, this adjustment brings the mean z-score of most of the physicians very close to 0, meaning that most of the variance between physicians is attributable to the sonographers whose exams they are interpreting and that the physicians themselves are not generally driving the measurements higher or lower.

Physician 5 is an outlier, with an adjusted mean z-score of 0.09, significantly farther from 0 than the other physicians. A likely explanation is that this physician systematically changed the sonographers’ numbers, either by selecting a larger AC than the sonographer selected or by remeasuring the AC and entering a larger number. A deviation of 0.09 SD is unlikely to be detected by image audit. Our approach is to review the results with the physician, ask how often they are changing the measurements, and point out that this may bias their results. A follow-up audit will confirm whether the issue persists.

## 4. Discussion

### 4.1. Quantitative Analysis and Focused Image Audits

The methods outlined fulfill the SMFM recommendation that QA for prenatal ultrasound should include both large-scale and focused audits [16]. For large-scale audits, we use the quantitative analysis of an entire practice for an entire year using standard parametric statistical techniques. For a focused audit, we perform image review for sonographers with outlier mean values. This approach avoids the time, complexity, and expense of performing image reviews for the majority of sonographers whose measurements fall within the expected range. 

Beyond mean values, we provide examples of three other issues that can be identified in the practice-wide quantitative analysis: (1) a standard deviation of z-score (SD) less than 1 suggests “expected-value bias” [21]; (2) a large SD suggests inconsistency in technique; and (3) physicians whose adjusted mean z-scores differ significantly from 0 may be systematically overriding sonographer measurements. These issues cannot easily be evaluated by image audit, but discussion with the involved providers may yield insights into areas for improvement.

### 4.2. Alternative Approaches

An entirely different approach to quality review is the RADPEER program of the American College of Radiology [22], in which a percentage of examinations are randomly selected for retrospective review by a second physician. The program was primarily designed for radiologic exams, but it can also be applied to ultrasound exams [18]. The RADPEER “double-reading” approach is time-consuming; for example, performing a review of 5% of just five types of radiological exams would require over 60 h per year for a skilled radiologist [23]. We are not aware of studies that assess the time requirements to double-read ultrasound exams. In RADPEER studies, the rates of discrepancy between the initial reading and the second reading are typically less than 10%, and most of these are judged not to be clinically significant [17,19,23]. In one study, the rate of significant discrepancy for ultrasound exams was less than 1% [18]. An important limitation of the RADPEER program is that the reviews are not blinded, so there is a potential for bias if, for example, the auditor is a subordinate of the person being reviewed [19]. Finally, the program evaluates only the reading physician, not the sonographer or radiology technician who obtains the images. 

Another approach to the quality control (QC) monitoring of fetal biometry measurements was adopted by the INTERGROWTH-21st project for its international prospective study of fetal growth [24]. For QC, each sonographer self-rated the quality of each biometry image using a standardized rating scale. Then, a random 10% sample of exams was selected for reevaluation by a central quality unit. There was a high level of agreement between sonographers and the central unit on both the qualitative assessment (kappa statistics: 0.99 for HC, 0.98 for AC, and 0.96 for FL) and the measurements (interobserver limits of agreement: ±4.4%, ±6.0%, and ±5.5%, respectively). The authors concluded that qualitative and quantitative QC are feasible and highly reproducible. They recommended these methods for both future research studies and clinical practice. We agree that comparable methods should be applied to well-funded research, but they may be too time-consuming and labor-intensive (and therefore expensive) to incorporate into routine clinical practice. 

### 4.3. Biometry Quality Review in Context

The review of biometric measurements is only one component of a comprehensive quality program for prenatal ultrasound. Other components include a review of fetal anatomy imaging and diagnostic accuracy. In forthcoming articles, we will describe our quantitative approach to the evaluation of performance on the fetal anatomy survey and the accuracy of fetal weight and sex determination. 

Beyond a review of the examination results, there are several structural components generally recommended for a high-quality ultrasound practice [15,16]. These include accreditation of the practice by an organization such as AIUM [13] or ACR [14]. Accreditation, in turn, requires that all personnel have adequate formal education and training in the theory and practice of the types of ultrasound exams performed by the practice, have certification by the appropriate body, and have several hours of continuing education annually. Accreditation also requires the practice to have written protocols to ensure the uniformity of exams, the timely interpretation and communication of findings, the disinfection and cleaning of transducers, the maintenance of equipment, and patient safety and confidentiality. Practices should also have a protocol for onboarding new sonographers and physicians that includes a formal orientation to practice protocols and a formal assessment of competency in the performance of various types of examinations [15].

### 4.4. Strengths and Limitations

A strength of the quantitative summary (Table 1 and Table 2) is that it provides a large-scale overview of an entire practice for a full year. Once the Stata script is written, the entire process requires only a few minutes each year to export the data and run the analysis. The method readily detects outlier sonographers and physicians for focused review. Analyzing a large number of exams for each provider, the method is highly sensitive to small variations.

An important limitation is the assumption that each sonographer performs exams on a random subset of patients. Each practice needs to carefully evaluate this assumption in order to properly interpret results. An example of a non-random selection might be a sonographer who only works on Tuesdays and Thursdays, which are, coincidentally, the 2 days when the practice sees and scans all the patients with diabetes, patients with a high rate of LGA fetuses; this sonographer would be expected to have a high z-score for AC even with perfect measurement. Another example might be a new-hire sonographer who has not yet been approved to scan patients with a body mass index over 30 kg/m^2^, another risk factor for LGA fetuses; this sonographer would be expected to have a lower-than-average z-score for AC. 

There are several other potential limitations. First, we do not adjust the analysis to account for the specific ultrasound machine or transducer used, which may introduce some bias into the measurements if certain machines have inaccuracies. Second, our analyses exclude multifetal gestations, precluding the ability to determine whether individual sonographers might have systematic errors in such pregnancies. However, multifetal pregnancies account for <5% of our exams, so the number of exams performed by each sonographer is usually too small for meaningful evaluation. Third, we do not adjust the analysis to account for patient race or ethnicity. A large multicenter study from the USA reported differences in fetal biometry between different race groups [25], but these were generally small. Despite those differences, the authors of that study subsequently published a set of unified standards applicable to all races [26]. Moreover, a high percentage of our patients decline to report race and ethnicity. Fourth, we did not exclude cases with fetal anomalies. Although there are some anomalies that might affect AC, for example, fetal hydrops, intraabdominal masses, or abdominal wall defects, these occur in <1% of fetuses and would therefore have a negligible impact on the mean z-score of personnel performing a reasonable volume of exams. As a practical matter, Viewpoint does not have a specific global field for fetal anomalies, so it would add considerable complexity and time (and therefore, expense) for us to identify and exclude such cases. Nonetheless, if a practice has the ability to easily identify cases with relevant anomalies, it would be preferable to exclude them. 

An important consideration is that high numbers of exams can result in very low *p*-values even with very small differences in z-score, that is, differences may be statistically significant even though they are too small to be clinically relevant and too small to be detected by image audit. Figure 1, panels B and C show an example of how difficult it is to see differences in an AC of 0.2 SD, corresponding to differences in a z-score of 0.2. As a “rule-of-thumb”, we find that image audits are generally not useful unless a sonographer’s z-score differs from the practice mean by at least 0.3. This criterion was met for Sonographers 1, 2, and 8 in Table 1. 

### 4.5. Future Directions

A major barrier to regular quality review is that personnel time must be dedicated to it, time that is not compensated by payers. The techniques we describe for the quantitative audit can be adopted with a few hours of development time by a person skilled with statistical software to adapt the analysis script. Once implemented, it takes less than an hour annually to export the data and run and interpret the analyses. Blinded image audits and the provision of feedback to providers take considerably more time.

A preferred alternative would be for developers and vendors of commercial ultrasound reporting software to include a suite of tools that would allow practices to readily summarize and compare z-scores for all the personnel in the practice for a variety of measurements, including basic biometry, special biometry, EFW, and other measurements. The tools should also include methods to generate a random sample of exams for a focused audit, as well as tools to readily identify and review extreme outlier observations. 

Some modern ultrasound systems are capable of using artificial intelligence (AI) to detect image planes and perform fetal biometry measurements. We hypothesize that the use of AI should reduce or eliminate between-sonographer variance in measurements. This hypothesis warrants testing as AI becomes increasingly adopted.

## 5. Conclusions

The large-scale quantitative analysis provides an overview of the biometry measurements of all the sonographers and physicians in a practice so that image audits can be focused on those whose measurements are outliers. The method also identifies several distinct types of systematic errors that image review alone would be unlikely to identify. The analysis takes little time to perform after initial development and avoids the time, complexity, and expense of auditing providers whose measurements fall within the expected range. We encourage commercial software developers, including those using AI for fetal biometry measurements, to include tools in their ultrasound reporting software to facilitate such quantitative reviews.

## Figures and Tables

**Table 1:** Abdominal circumference z-scores from selected sonographers.

Sonographer Number	Number of Exams	Mean z-Score	Standard Deviation of z-Score	Exams < 10th Percentile, n (%)	Exams > 90th Percentile, n (%)
1	116	−0.05	0.84	7 (6%)	6 (5%)
2	896	−0.03	1.02	48 (5.4%)	86 (9.6%)
3	1367	0.11 ^a^	1.11	100 (7.3%)	161 (11.8%)
4	1248	0.29 ^a,b^	1.12	73 (5.9%)	207 (16.6%)
5	1365	0.33 ^a,b^	1.09	66 (4.8%)	209 (15.3%)
6	182	0.52 ^a,c^	1.11	5 (3%)	41 (22.5%)
7	76	0.52 ^a,d^	1.35	1 (1%)	15 (19.7%)
8	972	0.63 ^a,e^	1.09	20 (2.1%)	236 (24.3%)
Total Practice	— ^f^	0.33 ^g^	1.08	4.6%	15.9%

^a^—Individual sonographer mean significantly different than 0 (*p* < 0.001, *t*-test); ^b^—Significantly larger than Sonographers 2 and 3 (*p* < 0.02, Scheffe test); ^c^—Significantly larger than Sonographers 1, 2, and 3 (*p* < 0.01, Scheffe test); ^d^—Significantly larger than Sonographer 2 (*p* < 0.02, Scheffe test); ^e^—Significantly larger than Sonographers 1 through 5 (*p* < 0.001, Scheffe test); ^f^—Total Practice includes sonographers tabulated plus others not shown. N suppressed to keep practice identity confidential; ^g^—Significant differences between sonographers overall (one-way ANOVA, *p* < 0.001).

**Table 2:** Abdominal circumference z-scores from selected physicians.

		Unadjusted		Adjusted for Sonographer	
Physician Number	Number of Exams	Mean z-Score	Standard Deviation of z-Score	Mean z-Score	Standard Deviation of z-Score
1	1348	0.17 ^a^	1.06	−0.05	1.04
2	4739	0.26 ^a^	1.06	0.00	1.04
3	2335	0.30 ^a^	1.10	−0.02	1.09
4	2090	0.44 ^a^	1.12	0.03	1.11
5	2044	0.57 ^a^	1.06	0.09 ^a,b^	1.07
Total Practice	--- ^c^	0.33 ^a^	1.08	0.00	1.07

^a^—Mean significantly different than 0 (*p* < 0.001, *t*-test); ^b^—Mean significantly different than Physicians 1, 2, and 3 (*p* < 0.05, Scheffe test); ^c^—N suppressed to keep identity of practice confidential.

## Data Availability

For investigators wishing to develop their own analysis script, we provide a supplementary Excel .XLSX file containing pseudo-data with 885 observations.

## Supplementary Materials

The following supporting information can be downloaded at: https://www.mdpi.com/article/10.3390/jcm13164860/s1. File S1: Sample Stata script for analysis (PDF) and Table S1: sample Excel file (XLSX) with pseudo-data for 885 exams.
