# Supplementary material for: Quantitative Approach to Quality Review of Prenatal Ultrasound Examinations: Fetal Biometry
Source: J Clin Med. 2024 Aug 17;13(16):4860. doi: 10.3390/jcm13164860 (PMC11355637; doi:10.3390/jcm13164860)
Supplement: Supplementary file 1 [file jcm-13-04860-s001.zip › File S1. DO-FILE QR Biometry Sample Script.pdf]

```
**** SAMPLE STATA SCRIPT FOR ANALYSIS OF FETAL ABDOMINAL CIRCUMFERENCE
* Supplement to "Quantitative approach to quality review of prenatal ultrasound
examinations - fetal biometry"
* Lines that start with an Asterisk (*) are comments
* Lines that start without an Asterisk  are Stata commands

cd  "/Users/andrewcombs/Desktop/Analysis/Approach to QR Sample script/"
log using "LOG Biometry QR Sample Script", replace
* log is a Stata file that keeps a listing off all comments, commands, and results.
* cd means Change Directory, making this the default file folder for data and results
* You will NEED TO CUSTOMIZE the default directory to match your computer's file structure

cd  "/Users/andrewcombs/Desktop/Analysis/Approach to QR Sample script/"
version 13
set more off

**** IMPORT EXCEL DATA FILE
import excel "EXCEL SiteName Year.xlsx", firstrow clear
* You will NEED TO CUSTOMIZE the name of the Excel file to match your file name

*********  DATA DICTIONARY ************
* The Excel file imported has the following fields (columns), named in Viewpoint:
*   Examdate - self explanatory
*   Examtype - examples Comp Basic, Comp Detailed, Follow up, Fetal Echo, etc
*   ExamID - arbitrary number assigned by Viewpoint, unique to each exam
*   Cardiacactivity - fetal heart beat (present or absent)
*   Numberofgestationalsacsfetu - 1=singleton, 2=twin, etc.
*   Sonographer - Viewpoint shows actual sonographer name.
*   Readingphysician - Viewpoint shows actual physician name
*         For confidentiality, we replaced sonographer and physician names
*         with dummy names.  For example, Sono-2 or Phys-3.
*         You NEED TO CUSTOMIZE with actual names any place you see these dummy names.
*         (Dummy names are used in the graph commands)
*   AssignedEDD - "due date" based on best obstetrical estimate
*   BPDmm - biparietal diameter in mm
*   HCmm - head circumference in mm
```

```
*   ACmm - abdominal circumference in mm
*   Femurmm - femur length in mm

*** DEFINE NEW VARIABLES and CLEAN-UP
generate GAd = 280 - (AssignedEDD - Examdate)
generate GA  = GAd/7
*  GAd is gestational age in days
*  GA is gestational age in weeks

generate HC=HCmm/10
generate AC=ACmm/10
generate FL=Femurmm/10
* HC, AC, FL are measurements in centimeters

keep if (trim(Examstatus)=="Finalized" | substr(trim(Examstatus),1,8)=="Revised ")
keep if trim(Numberofgestationalsacsfetu) == "1"
keep if trim(Cardiacactivity) == "present"
keep if GA>=14 & GA<41
* These steps will drop incomplete reports, multifetal pregnancy, absent fetal heart beat
*    and GA outside target range 14-40 weeks.


*** CALCULATE z-SCORE FOR AC
generate hadAC= -13.3 + 1.61*GA - 0.00998*GA^2
generate sdAC=1.34
generate zAC= (AC-hadAC)/sdAC
* hadAC is Hadlock's predicted AC for gestational age.
* The formula is in Hadlock et al, Radiology 1884;152:497-501, footnote to Table III
* sdAC is Hadlock's standard deviation of AC, same source, constant at 1.34 cm
* zAC is calculated z-Score.

list Examdate, ExamID, GA, AC, zAC if abs(zAC) >6
* lists extreme outliers (absolute value of zAC more than 6)
drop if abs(zAC) >6
* deletes extreme outliers
```

```
*** CALCULATIONS FOR TABLE 1: z-SCORES BY SONOGRAPHER
oneway zAC Sonographer, tabulate scheffe
* oneway is the Stata command for oneway ANOVA
* The tabulate option generates a table similar to Table 1 in our article
*     (columns for number of exams, mean z-score, and SD of z).
* At the bottom of the displayed table, the value under "Prob >F" is overall P-value.
* A P-value < 0.05 means that some of the songraphers have a different mean z-score
*    than others, but does not show who are different.
* The scheffe option generates a multiple comparisons table, showing P-values for
*     all possible 2-way comparisons between sonographers.
summarize zAC
* summarize shows the mean and SD of z-score for the entire practice
* (includes sonographers not included in Sono-1 through Sono-8)

bysort Sonographer: ttest zAC==0
* the t-test assesses whether mean is different than 0

* the next section generates values for the right 2 columns of Table 1
generate zClass=""
replace zClass="LGA" if zAC<.
replace zClass="AGA" if zAC<=1.2816
replace zClass="SGA" if zAC<-1.2816
* z-score  1.2816 is the 90th percentile
* z-score -1,2816 is the 10th percentile
tabulate Sonographer zClass, row chi
tabulate zClass


*** CALCULATIONS FOR TABLE 2: z-SCORES BY PHYSICIAN

oneway zAC Readingphysician, tabulate
* oneway ANOVA of AC z-score, unadjusted columns of Table 2 (mean and SD)

* adjust z-score for sonographer
replace Sonographer="Other " if Sonographer==""
bysort Sonographer: egen SonoMean=mean(zAC)
```

```
generate zACadj = zAC-SonoMean
* SonoMean is each sonographer's mean z-score
* zACadj adjusts each observed z-score by subtracting songrapher mean
oneway zACadj Readingphysician, tabulate scheffe
* this ANOVA is used for the Adjusted columns of Table 2.
bysort Readingphysician: ttest zACadj==0
* the t-test is used to assess whether mean is different than 0


*** GENERATE LIST OF 20 EXAMS for IMAGE REVIEW

* You NEED TO CUSTOMIZE the "generate randomID" command: insert sonographer name instead of
"Sono-2" etc.
* The "runiform()" function generates a random number between 0 and 1.
* tGA is GA in completed weeks (truncated by removing any decimal fraction of weeks).
* After sort, the exams are in numerical order from smallest to largest randomID.
* Then we list the first 20 exams.

generate randomID=runiform() if Sonographer=="Sono-2"
generate tGA=trunc(GA)
sort randomID
list ExamID Examdate Examtype tGA Sonographer in 1/20, noobs

* Repeat the process for another sonographer.
* Again, you NEED TO CUSTOMIZE by inserting sonographer name rather than "Sono-8"
drop randomID
generate randomID=runiform() if Sonographer=="Sono-8"
sort randomID
list ExamID Examdate Examtype tGA Sonographer in 1/20, noobs

* Copy-paste these 4 commands as needed to add listings for other sonographers.



*********** GRAPHS *********
```

```
*** FIGURE 2 SCATTERPLOTS AND HISTOGRAMS

* You NEED TO CUSTOMIZE these commands: insert sonographer name instead of "Sono-2" etc.
* For the "graph save" commands, you can insert your own title insetead of "Sono-2"
* The /// at the end of some lines means that the command continues on the next line.

generate AC90=hadAC + 1.2816*sdAC
generate AC10=hadAC - 1.2816*sdAC
bysort GA: generate nGA=_n

twoway (scatter AC GA if(Sonographer=="Sono-2"), mcolor(blue) msymbol(smx)) ///
    (line AC90 hadAC AC10 GA if(nGA==1), lcolor(red black green)), ///
    ytitle(Abdominal Circumference mm) xtitle(Gestational Age weeks) ///
    legend(position(11) ring(0) label(1 Sonographer 2) label(3 AC50))
graph save Graph "GRAPH zAC scatterplot tech 2.gph", replace

twoway (scatter AC GA if(Sonographer=="Sono-8"), mcolor(blue) msymbol(smx)) ///
    (line AC90 hadAC AC10 GA if(nGA==1), lcolor(red black green)), ///
    ytitle(Abdominal Circumference mm) xtitle(Gestational Age weeks) ///
    legend(position(11) ring(0) label(1 Sonographer 8) label(3 AC50))
graph save Graph "GRAPH zAC scatterplot tech 8.gph", replace

histogram zAC if zAC^2 <16 & Sonographer=="Sono-2", xtitle("AC z-score, Sonographer-2") ///
    addplot(function y=normalden(x), range(-4 4) droplines(-1.2816 0 1.2816)) legend(off)
graph save Graph "GRAPH zAC Sono-2.gph" , replace

histogram zAC if zAC^2 <16 & Sonographer=="Sono-8", xtitle("AC z-score, Sonographer-8") ///
    addplot(function y=normalden(x), range(-4 4) droplines(-1.2816 0 1.2816)) legend(off)
graph save Graph "GRAPH zAC Sono-8.gph" , replace


**** FIGURE 3 - COMBINING 2 NORMAL CURVES WITH DIFFERENT MEANS and SD=1
cd  "/Users/andrewcombs/Desktop/Analysis/Approach to QR Biometry/Graphs"
generate Even = 0
replace Even=1 if ExamID/2==trunc(ExamID/2)
generate Nml=.
```

```
replace Nml=rnormal()-0.5 if Even==1
replace Nml=rnormal()+0.5 if Even==0
*  The rnormal() function generates random number that are normally distributed.
*  This amounts to a Montecarlo simulation of random normal sampling from the population
*  If the ExamID is an even number, the function generates a normal distribution with z =
-0.5
*  If the ExamID is odd, the function generates a normal distribution with z = +0.5

histogram Nml if Nml^2 <16 & Even==1, xtitle("z-score, fetuses w risk of SGA") ///
    addplot(function y=normalden(x), range(-4 4) droplines(-1.2816 0 1.2816)) legend(off)
graph save Graph "GRAPH z SGA.gph" , replace

histogram Nml if Nml^2 <16 & Even==0, xtitle("z-score, fetuses w risk of LGA") ///
    addplot(function y=normalden(x), range(-4 4) droplines(-1.2816 0 1.2816)) legend(off)
graph save Graph "GRAPH z LGA.gph" , replace

histogram Nml if Nml^2 <16, xtitle("z-score, combined") ///
    addplot(function y=normalden(x), range(-4 4) droplines(-1.2816 0 1.2816)) legend(off)
graph save Graph "GRAPH z Both.gph" , replace


**** FIGURE 4 - HISTOGRAMS FOR SONOGRAPHER 3 and SONOGRAPHER 1

histogram zAC if zAC^2 <16 & Sonographer=="Sono-3", xtitle("AC z-score, Sonographer-3") ///
    addplot(function y=normalden(x), range(-4 4) droplines(-1.2816 0 1.2816)) legend(off)
graph save Graph "GRAPH zAC Sono-3.gph" , replace

histogram zAC if zAC^2 <16 & Sonographer=="Sono-1", xtitle("AC z-score, Sonographer-1") ///
    addplot(function y=normalden(x), range(-4 4) droplines(-1.2816 0 1.2816)) legend(off)
graph save Graph "GRAPH zAC Sono-1.gph" , replace

**** z-SCORES FOR HC and FL
*   Hadlock 1984, same reference as for AC, formulas in footnote to Table III.
*   Analysis can use same scripts as for AC, replacing "AC" with "HC" or "FL" everywhere.

*** HC
```

```
generate hadHC=-11.48+ 1.56*GA - .0002548*GA^3
generate sdHC=1
generate zHC=(HC-hadHC)/sdHC

*** FL
generate hadFL= -3.91 + 0.427*GA - 0.0034*GA^2
generate sdFL = 0.3
generate zFL = (FL-hadFL)/sdFL

save "DATA from Sample Script.dta", replace
log close
exit
```
